# Human Cadaveric Artificial Lung Tumor-Mimic Training Model

**DOI:** 10.3389/pore.2021.630459

**Published:** 2021-04-26

**Authors:** Réka Székely, Ferenc Imre Suhai, Kinga Karlinger, Gábor Baksa, Bence Szabaczki, László Bárány, Gergely Pölöskei, Gergely Rácz, Ödön Wagner, Béla Merkely, Tamás Ruttkay

**Affiliations:** ^1^ Laboratory for Applied and Clinical Anatomy, Department of Anatomy, Histology and Embryology, Semmelweis University, Budapest, Hungary; ^2^ Heart and Vascular Center, Semmelweis University, Budapest, Hungary; ^3^ Medical Imaging Center, Semmelweis University, Budapest, Hungary; ^4^ 1st Department of Pathology and Experimental Cancer Research, Semmelweis University, Budapest, Hungary; ^5^ Department of Inorganic and Analytical Chemistry, Budapest University of Technology and Economics, Budapest, Hungary

**Keywords:** tumor mimic, pseudotumor, cadaver workshop, hands-on training, lung tumor

## Abstract

**Introduction:** An important phase in surgical training is gaining experience in real human anatomical situations. When a cadaver is available it may complement the various artificial practice models. However, it is often necessary to supplement the characteristics of the cadavers with a simulation of a tumor. Our objective was to develop an easy-to-create, realistic artificial tumor-mimic model for peripheral lung tumor resection practice.

**Methods:** In our work we injected barium sulphate enriched silicone suspension into 10 isolated, non-fixed lungs of human cadavers, through the puncture of the visceral pleura. Four lesions–apical, hilar and two peripheral–were created in each of ten specimens. After fixation CT scans were obtained and analyzed. The implanted tumor-mimics were examined after anatomical preparation and slicing. Also performed CT-guided percutaneous puncture was also performed to create the lesions *in situ* in two lungs of human cadavers.

**Results:** Analyzing the CT data of 10 isolated lungs, out of 40 lesions, 34 were nodular (85.0%) and in the nodular group five were spiculated (12.5%). Satellite lesions were formed in two cases (5.0%). Relevant outflow into vessels or airway occurred in five lesions (12.5%). Reaching the surface of the lung occured in 11 lesions (27.5%). The tumor-mimics were elastic and adhered well to the surrounding tissue. The two lesions, implanted *via* percutaneous puncture, both were nodular and one also showed lobulated features.

**Conclusion:** Our artificial tumor-mimics were easy to create, varied in shape and size, and with percutaneous implantation the lesions provide a model for teaching every step of a surgical procedure.

## Introduction

Proper learning of the skills of thoracic surgery is one of the significant challenges for an aspiring surgeon. There are several reasons for that including the surgeon ability to reliably handle endoscopic manipulations, to apply airtight sutures and to remove target lesions with adequate healthy tissue margin. The collapse of the lung adds difficulty as it significantly changes the anatomy in the intraoperative situation, compared to the anatomy seen in the preoperative diagnostic imaging. Planning the removal of the target lesion, finding it during the procedure, and resecting successfully, are complex tasks requiring proper theoretical knowledge and practical experience. The possibilities to obtain the required *ex vivo* experience however are quite limited [1–4].

Artificial models, mimicking different types of tumors were developed by others. The use of these tumor-mimics have multiple purposes as they are used for testing imaging modalities, developing ultrasound localization, radiofrequency ablation devices and surgical navigation systems and they can also be used in surgical training. To create artificial lesions those have similar characteristics to real tumor tissue echogenicity and have similar ablation attributes, agarose [5–9] or fluid, composed of purified bovine serum albumin and glutaraldehyde [10] were used in various organs. Others used gelatin [8, 9, 11] chromatic alginate [11], ultrasound gel [12], paste made of animal muscle tissue [13–15] or liquid plastics [16]. Viable tumor cells were transplanted into living animals [17, 18] for testing models and real lesion morphology was imitated with implanted lesions made from polymer [19, 20]. 3D printed models [21, 22] of artificial tumor-mimics included their surrounding tissue and the whole organ. Most of these lung related models [5, 8, 9, 13–15, 17, 18] that were developed for testing, are suboptimal for surgical training.

Our aim was to develop a model for educational use, a model, which is easy to create and can imitate the real tumor morphology in the lung. The size and location of the artificial tumor-mimics may be selected for the planned intervention, and can be adapted to the particular teaching conditions.

## Materials and Methods

In part of our investigation we implanted artificial tumor-mimics into non-fixed five right and five left lungs, that were originated from six human cadavers, and were removed in accordance with proper autopsy procedure. The cadavers were donated to the institution of the first author for the purpose of medical education and research.

**FIGURE 1:**
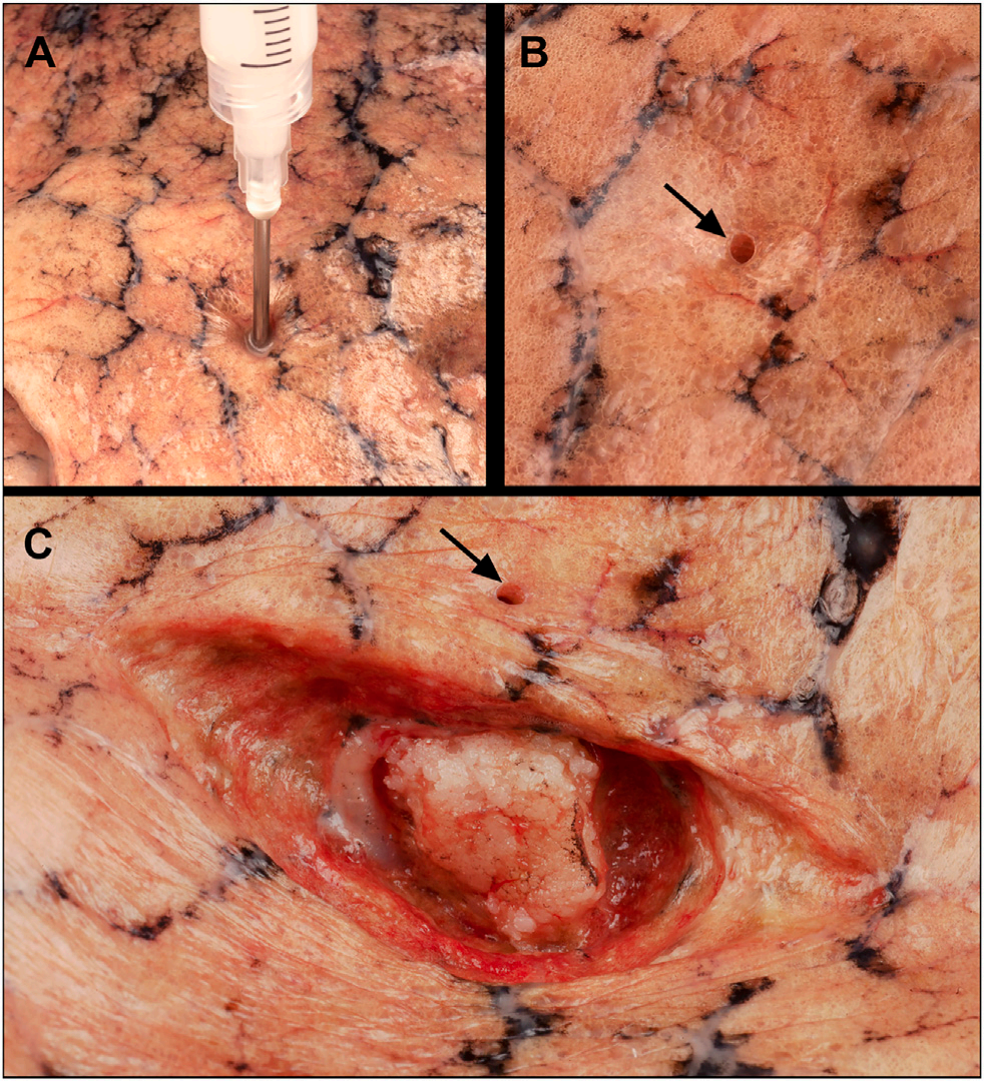
Injection of the silicon tumor model into a removed non-fixed cadaveric lung. **(A)** Puncture through the visceral pleura, a 15G needle inserted perpendicular to the surface, approximately 15 mm deep; **(B)** Surface of the lung showing the hole created by the needle puncture (black arrow); **(C)** Exposing the artificial tumor through a longitudinal incision of the lung parenchyma, black arrow: hole.

The lesions were created by injecting a silicone suspension into the lung tissue. The suspension was produced using 20 w/w% barium sulphate contrast agent (Merck KGaA, Darmstadt, Germany) and 50–75 w/w% condensation type of polydimethyl-siloxane elastomer (R-5000 type, T-Silox Ltd., Hungary); 0–25 w/w% silicon oil (M350 type, T-Silox Ltd., Hungary) and 5 w/w% networking agent (Oxam, T-Silox Ltd., Hungary) were used to get the optimal consistency. The suspension was delivered to the desired location by injecting it through a puncture of the visceral pleura (Figures 1A–C). To enhance visibility of the transparent silicone and the white barium sulphate (Figure 1C) we added yellow dye (Bayferrox 920; KLORID; Püspökladány, Hungary) in one lung, brown dye (Ferric oxide; KLORID; Püspökladány, Hungary) in three lungs and green dye (Uvo Green; Smooth-On; Easton, PA, United States) in two lungs. Four lesions were created in each lung at different locations, one apical (injection of 4.0 ml silicone suspension), one hilar (injection of 2.0 ml silicone suspension) and two peripheral (injection of 1.0 ml and 0.5 ml silicone suspension into the middle or inferior lobe). The collapsed lungs were injected with a 4 cm long 15G needle (attached to 2 or 10 ml syringes) approximately 15 mm deep under the visceral pleura, holding the needle perpendicular to the lung surface (Figure 1A). The needle was kept in place until the onset of the hardening of the silicone mixture.

Then the main bronchus was cannulated with a 10 mm diameter silicone tube and was connected to a diaphragm pump (air 275 R plus; sera; Heinsberg, Germany). The preparations were insufflated, increasing the pressure in small increments until it reached 24 mbar. To the bottom of a closed plastic box 500 ml formaldehyde in 37% aqueous solution was added, the lungs and the diaphragm pump were suspended above the liquid. Filling the closed space with recirculated formalin vapor and insufflating the lung with formaldehyde air were done to preserve the tissue for optimal assessment of the location and morphology of the created artificial tumors. The fixation was maintained for 7 days at 25°C.

**FIGURE 2:**
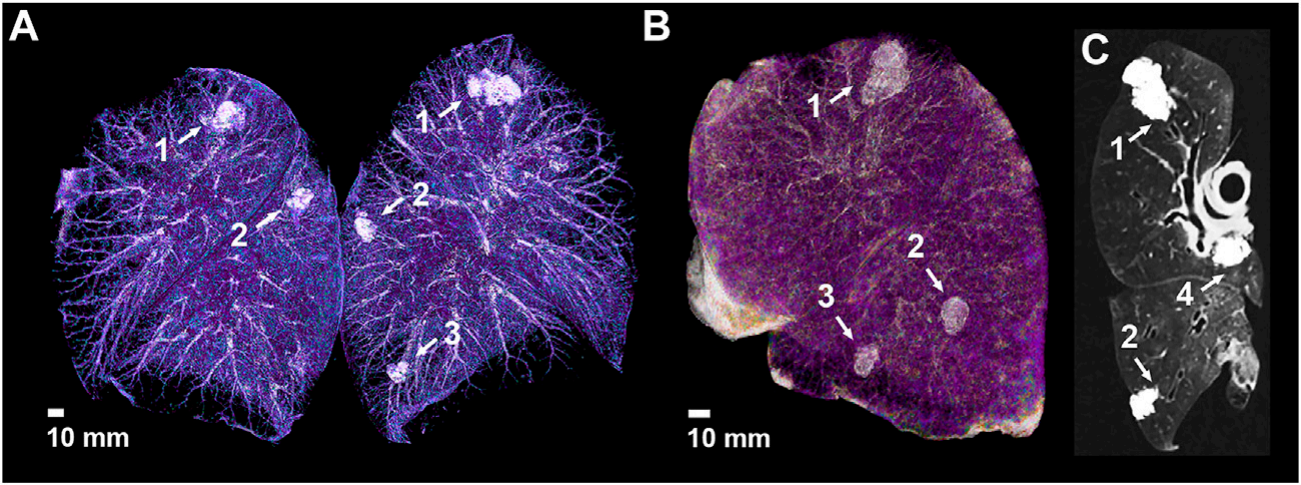
CT imaging of the isolated lungs implanted the artificial tumors. **(A)** Volume rendering of the CT scan with artificial tumors in different locations of both lungs; **(B)** Anterolateral view of a left lung showing three of the implanted artificial tumors; **(C)** Oblique CT slide of the left lung showing the fourth hilar lesion– 1: apical lesion, 2: 1.0 ml peripheral lesion, 3: 0.5 ml peripheral lesion, 4: hilar lesion.

**FIGURE 3:**
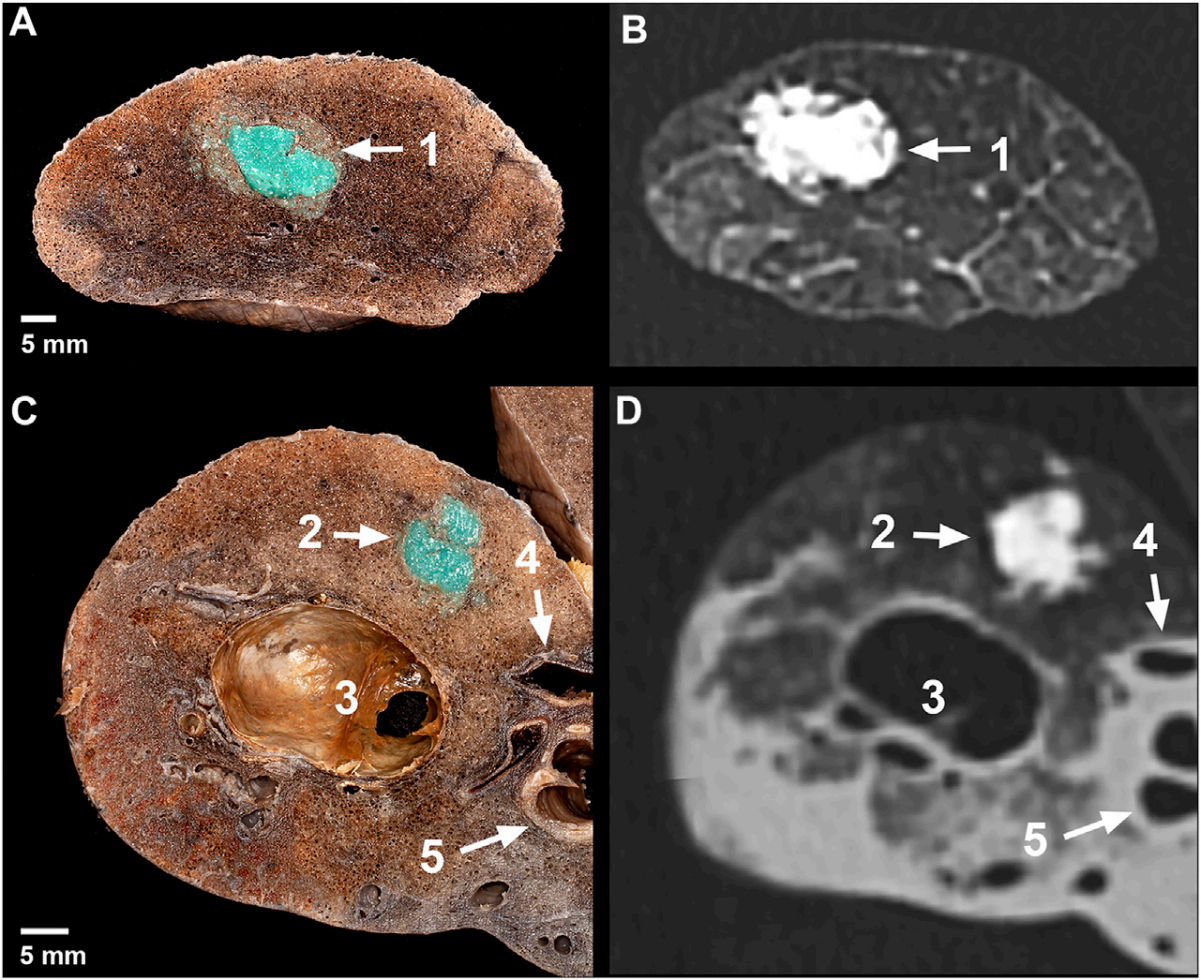
Macroscopic photographs and CT images of the artificial tumors. Axial slice **(A)** and its CT image **(B)** in the apical region of a right lung– 1: apical lesion; Axial slice **(C)** and its CT image **(D)** in the middle lobe of a right lung– 2: 1.0 ml peripheral lesion with spiculated formation, 3: emphysematous bulla, 4: medial segmental artery, 5: lateral segmental bronchus.

After fixation, helical CT scanning was performed on each prepared lung in cranio-caudal direction using a 256-slice CT scanner (iCT 256; Philips Healthcare; Best, Netherlands; tube voltage 120 kV, tube current 30 mAs). The images were acquired with 0.9 mm slice thickness, 0.45 mm increment, and were reconstructed using iterative reconstruction (iDose4) (Figures 2A–C and Figures 3B,D). In connection with the spatial extent of the tumors, three diameters per lesion were measured, and volumes were calculated. The measurements were taken by an experienced radiologist, using a lung window setting on the displayed CT scan as follows: in the axial plane measured the largest size, that determines a direction (direction A), measured the size perpendicular to the direction of the largest size in each lesion (direction B) and the largest cranio-caudal size (direction C). Based on the CT scans, the artificial tumors were classified according to their shape (nodular, tubular, lobulated, spiculated), and whether satellite lesion occurred, came into contact with visceral pleura, air bubble formed in them, and streamed into lumens of surrounding blood vessels or airways.

**FIGURE 4:**
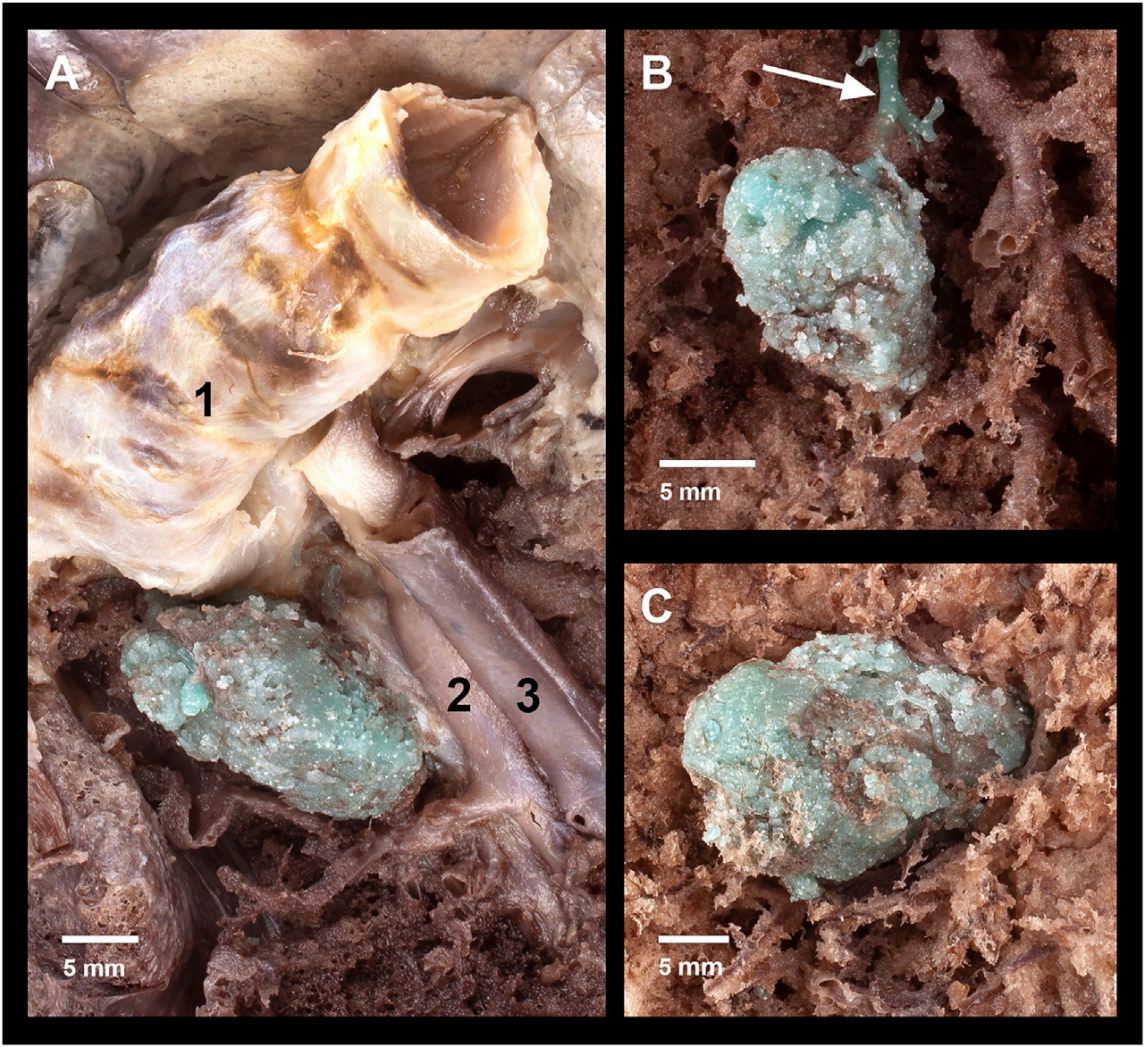
Anatomic preparations of the artificial tumors. **(A)** Region of a hilar lesion– 1: left main bronchus, 2: lingular artery, 3: lingular vein.; **(B)** Region of a 0.5 ml peripheral lesion– white arrow: efflux into a peripheral branch of left inferior pulmonary vein; **(C)** Region of a 1.0 ml peripheral lesion.

The isolated lung specimens were manually prepared and sliced. The artificial lesions were revealed by preserving the significant surrounding structures (blood vessels, bronchus). Four apical, three hilar (Figure 4A), four 1 ml peripheral (Figure 4C) and five 0.5 ml peripheral (Figure 4B) lesions were prepared. Six apical (Figure 3A), seven hilar, six 1.0 ml peripheral (Figure 3C) and five 0.5 ml peripheral lesions of the lung tissue were cut axially into 5 mm thick slices.

**FIGURE 5:**
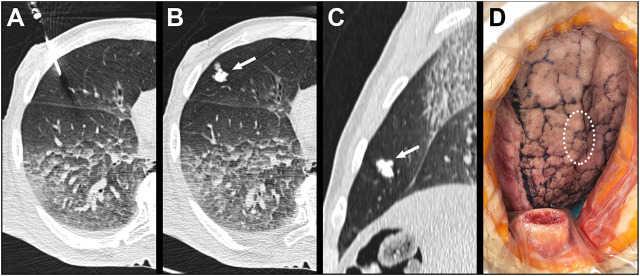
Implantation of the artificial tumor into an intact cadaveric chest through CT-guided percutaneous puncture. **(A)** CT scan shows the 14G needle puncturing the lung; **(B)** Axial view of the 1.0 ml lesion in the S V segment of the right lung; **(C)** Sagittal view of the 1.0 ml lesion; **(D)** Surface of the lung visible through right anterolateral thoracotomy– dotted line: the location of the palpable but not visible lesion.

During our investigation, artificial tumor-mimics were also implanted into the lungs of two non-fixed cadavers via CT-guided percutaneous puncture (Figure 5A). Helical CT scan was performed on the chest using a 64-slice CT scanner (Ingenuity Core 64; Philips Healthcare; Best, Netherlands; tube voltage 120 kV with tube current 167 mA and 100 kV and 150 mA). The images were acquired with 2 mm slice thickness, 1 mm increment, and were reconstructed using iterative reconstruction. No pathologically significant lesions were found in the chest during the initial native examinations. The percutaneous puncture was done with a 14G thick, 50 mm long needle in an approximate perpendicular position to the skin surface on the right side of the body, in the third intercostal space in the first case, and in the fourth intercostal space in the second case. Then 1.0 ml silicone suspension was injected into the parenchyma of the S IV (case 1) and S V (case 2) segments, 15 mm deep under the visceral pleura. The needle was kept in place until the onset of the hardening of the silicone mixture. In order to examine the artificially produced tumor-mimics, CT scan was acquired of the chest following the implantation (Figures 5B,C).

Subsequently we studied the surface of the lung segment that contained the lesion through a 100 mm long right anterolateral thoracotomy in the fourth intercostal space. In the second case part of the fourth rib was resected. (Figure 5D). Following endotracheal intubation, under visual control the lung was insufflated to 10, 20 and 30 mbar pressure, using the diaphragm pump.

## Results

Analyzing the CT scans of the 10 isolated lungs, each with artificial tumor-mimics injected into four different locations, the 40 artificial tumors were identified and also examined qualitatively and quantitatively. The summary of the measurements is detailed in Table 1.

**TABLE 1:** Measurements and qualities of the artificial tumors in isolated lungs.

Variables	Apical tumor	Hilar tumor	Peripheral 1.0 tumor	Peripheral 0.5 tumor	Total (*n* = 40)
Size in direction A (mm) mean ± SD (range)	23.00 ± 3.92 (19–33)	16.00 ± 4.22 (11–27)	14.80 ± 1.93 (12–19)	11.90 ± 3.45 (9–19)	
Size in direction B (mm) mean ± SD (range)	14.80 ± 1.48 (13–17)	11,10 ± 2.77 (7–15)	10.70 ± 1.95 (7–14)	7.30 ± 2.06 (4–11)	
Size in direction C (mm) mean ± SD (range)	24.00 ± 4,97 (18–33)	15.70 ± 3.40 (9–22)	14.30 ± 2.91 (10–18)	11.40 ± 1.84 (9–14)	
Volume (mm^3^) mean ± SD (range)	4220.69 ± 897.09 (3134–5391)	1472.73 ± 539.11 (415–2262)	1207.06 ± 418.72 (550–1994)	505.69 ± 187.17 (297–899)	
Formation, *n* (%)					
** **Nodular	8 (20.0)	8 (20.0)	6 (15.0)	6 (15.0)	28 (70.0)
** **Nodular, spiculated	1 (2.5)	0 (0.0)	2 (5.0)	2 (5.0)	5 (12.5)
** **Nodular, lobulated	0 (0.0)	1 (2.5)	0 (0.0)	0 (0.0)	1 (2.5)
** **Tubular	1 (2.5)	1 (2.5)	2 (5.0)	2 (5.0)	6 (15.0)
Pleural involvement, *n* (%)	6 (15.0)	2 (5.0)	1 (2.5)	2 (5.0)	11 (27.5)
Satellite lesion, *n* (%)	1 (2.5)	0 (0.0)	0 (0.0)	1 (2.5)	2 (5.0)
Air bubbles, *n* (%)	4 (10.0)	2 (5.0)	3 (7.5)	5 (12.5)	14 (35.0)
Efflux in vasculature or airway, *n* (%)	1 (2.5)	1 (2.5)	1 (2.5)	2 (5.0)	5 (12.5)

Tissue properties around the injected silicone suspension influenced the spread toward the lung surface. It was significant in 11 cases (27.5%) where the lesions reached the visceral pleura. In apical tumor-mimics, in six cases (15.0%) pleural involvement was most prevalent. Satellite lesion of the injected silicone material was observed in one apical (2.5%) and one peripheral 0.5 ml (2.5%) tumor-mimic. During the injection, in 14 cases (35.0%) air bubbles formed in the artificial tumor mass and were visible on the CT scans. Contrary to the peripheral location of the injected tumors near the lung surface, one apical (2.5%), one hilar (2.5%) and three peripheral (7.5%) lesions showed relevant efflux into the blood vessels or into the airway.

Predominantly nodular tumors (34, 85.0%) were formed, beside that tubular form was observed in six cases (15.0%). Among the nodular tumors five showed spiculated contours (12.5%) and one tumor had lobulated appearance (2.5%).

The largest dimension in apical tumors was in direction C (24.00 mm ± 4.97; 18–33). The largest measurement was in direction A in hilar lesions (16.00 mm ± 4.22; 11–27), 1 ml peripheral tumors (14.80 mm ± 1.93; 12–19) and 0.5 ml peripheral lesions (11.90 mm ± 3.45; 9–19) followed. The calculated lesion volume, in accordance with the injected amount of silicone material, was the largest in apical tumors (4220.69 mm3 ± 897.09; 3134–5391), then in descending order in hilar (1472.73 mm3 ± 539.11; 415–2262), 1 ml peripheral (1207.06 mm3 ± 418.72; 550–1994) and 0.5 ml peripheral (505.69 mm3 ± 187.17; 297–899) tumors.

The tumor-mimics created from the silicon suspension were elastic, robust and they did not easily tear to the touch, and adhered well to the surrounding tissue. The lesions containing the dye distinctly contrasted with the environment, therefore their shape and extent could be optimally examined (Figures 4A–C and Figures 3A,C). The morphology of the surgical tumor-mimics were identical to those described per the CT scans.

The preservation was appropriate for the examinations of the artificial tumors within the anatomical conditions of the inflated lung. After 7 days of fixation the consistency of the lung was still sponge-like, and resumed its inflated form after squeezing. Fixed tissue was found macroscopically after preparing and slicing the lung.

The percutaneous puncture was performed under CT-guidance, which helped to plant the artificial tumor at the desired location. The silicone suspension in the lung parenchyma behaved similarly as it did in the isolated lung (size in direction A: 17 mm and 20 mm, size in direction B: 7 mm and 7 mm, size in direction C: 14 mm and 10 mm, volume: 872 mm3 and 733 mm3). Both lesions showed nodule formation and one of them was lobulated and they did not show any satellite lesions or air bubbles. Both of them reached the visceral pleura, and in the first case it had an efflux into the airway and pulmonary vasculature. In one case local pneumothorax developed due to the percutaneous puncture. Both lungs were successfully ventilatable, no significant leakage was detected through the needle perforation.

## Discussion

There are many challenges in thoracic surgery. The collapse of the lung, due to air getting in between the parietal and the visceral plate of the pleura during the thoracotomy, has the most significant effect on the lung anatomy. Finding the lesions that are often small–found in preoperative imaging–requires extraordinary expertise. Gaining experience is invariably limited for resident surgeons [1–4].

In this study we created lung tumor models for surgical training and research. Artificial tumors produced by injecting silicone into the lung parenchyma with the help of CT-guidance provide an opportunity that the stages from diagnosis and planning of the operation to professional surgical intervention can be performed in human anatomical conditions.

Agarose tumor models have been developed in other parenchymal organs [6–8] and also in the lung [5] primarily for testing and practicing of radiofrequency ablation. After consolidation in the mold the tumor was implanted through a surgical incision that later was closed by a running suture. Others have used models made of agarose [9] for bronchoscopy training and similarly to our method, they have injected material under CT-guidance. The material we used is much more compact and palpable, less fragile, and does not disintegrate as much as soft agarose does. In addition, our artificial tumors made of silicone suspension showed realistic macroscopic properties on the CT scans, forming nodular and spiculated masses, and natural relationship to anatomic structures. Since the implantation procedure is done with percutaneous puncture, damage to the lung tissue is minimal, allowing the usage of ventilation. By maintaining the integrity of the chest, our model is also suited for surgical excision as every step of an interventional procedure can be carried out.

Muscle paste mixed with barium sulfate, made by others for radiofrequency ablation testing [13] and application [14, 15] was injected with a bone biopsy needle, guided by fluoroscopy. Producing muscle paste is time-consuming as it requires strictly controlled conditions, and muscle tissue removal from a live animal. After the intervention, histological examination is required to determine the success rate. Using viable tissue to evaluate necrosis can be an important factor to take into consideration for radiofrequency ablation, but in our case it is irrelevant. In our model the silicone mixture consists of inexpensive ingredients and it can easily be injected with a 15G needle into the lung parenchyma. After approximately 30 min of hardening the operation can be performed. Due to the contrasting appearance of the green colored artificial tumor, the success of the operation can be determined by visual cues, the evaluation does not require histological examination.

In a model of VX2 carcinoma [17] viable tumor cells were used and were implanted with CT-guided puncture. Implantation was done only on living animals and for that tumor donor animals were required. The time-consuming treatment of the living animals and tumor cells are expensive. The emergence of the tumor takes several weeks (in the referred publication 14–21 days) and the exact location of the lesion or any metastases cannot be influenced.

Polymers [19, 20] and the agarose models without contrast agents are suitable for neurosurgical simulations or when the lesion is plainly visible. Our artificial tumor-mimics contain a barium sulphate contrast agent; it enhances the visibility of the lesion on the CT scans, the thin extensions (spiculated formation) could be more accurately inspected, thereby providing the opportunity for realistic pre-surgical planning for radiologists and surgeons.

In conclusion, comparing our silicone suspension tumor model with the previously described models, the advantage are as follow: it can be prepared efficiently; the two inexpensive ingredients are easily combined and injected into the cadaver; the material solidifies in a short period of time; when injected into the lung parenchyma by percutaneous puncture the chest remains intact; and it does not interfere with surgical exploration. Furthermore, in appearance and consistency, our model imitates the presentation of the tumor both in medical imaging and in reality, after the model creation is completed the operation can be started immediately, long preparation time is not required. Additionally the model can be used in both fresh and preserved cadaver.

The limitation of our model is that the actual shape of the artificial tumor is determined by the surrounding morphology. The characteristic of the tissue surrounding the tip of the inserted needle determines the direction of the silicone suspension flow, where the high pressure of the injection will force it. It may spread within the parenchyma or become superficial, it can flow into a lumen, it may break out to the surface of the pleura along the puncture canal, it can take spherical or elongated shape and it can form protrusions.

In the process of gaining surgical experience every opportunity is invaluable. According to international literature the last and highest stage of medical education is considered to be the hands-on training on human cadavers, accepting the limited availability and associated cost. The real anatomical conditions may be complemented with reproducible tumor mimics to get a complex training model. While the obtained radiological result using our model is diverse, almost without exception, it imitates the types of tumors that occur in clinical settings. This method allows the participant not only to develop manual skills in a simplified model, but to practice a complete clinical case from the point of dosing to the tumor removal, based on guidelines.

## Data Availability

The raw data supporting the conclusions of this article will be made available upon request by the authors, without undue reservation.

## Abbreviations

CT, computed tomography; S IV, lateral segment of the middle lobe (right lung); S V, medial segment of the middle lobe (right lung).
